# Long‐Term Impact of the Largest Environmental Disaster in Latin America (Fundão Dam Failure) on Microbial Communities in Lakes of the Doce River Basin, Brazil

**DOI:** 10.1111/1462-2920.70171

**Published:** 2025-09-01

**Authors:** Pedro Almeida, André Torres, Marcelos Gomes, Ernesto Caffarena, Hugo Jesus, Pedro Pereira, Katariny Pereira Dos Santos, Carlos Eduardo Delfino Vieira, Yuri Dornelles Zebral, Camila Martins, Adalto Bianchini, Henrique Santos

**Affiliations:** ^1^ Departamento de Biologia Marinha Universidade Federal Fluminense Rio de Janeiro Brazil; ^2^ Programa de Computação Científica (PROCC), Fundação Oswaldo Cruz (Fiocruz) Rio de Janeiro Brazil; ^3^ Laboratório de Biologia, Controle e Vigilância de Insetos Vetores (LBCVIV) Instituto Oswaldo Cruz Rio de Janeiro Brazil; ^4^ Instituto de Ciências Biológicas, Universidade Federal Do Rio Grande – FURG Rio Grande Brazil

**Keywords:** 16S rRNA, co‐occurrence, heavy metals, lake microbiology, mine waste, water microbiology

## Abstract

The collapse of the Fundão tailings dam in 2015 stands as the largest environmental disaster in Latin America and the global mining industry. This catastrophic event released around 62 million m^3^ of mining waste into the Doce River basin. This study aimed to assess the long‐term effects of the disaster by analysing microbial communities in four lakes within the Doce River basin. Conducted over 4 years (2018–2021), with a total of six sampling campaigns. The microbiome of water and sediment was analysed using high‐throughput 16S rRNA gene sequencing. The results demonstrate a significant correlation between key microbial groups and metals associated with the disaster, including *Deinococcus*, *Thermoanaerobaculaceae*, *Pirellula*, and *Rhodospirillaceae*. Moreover, an enrichment of genes associated with xenobiotic degradation and metal metabolism pathways was detected. These findings suggest that microbial communities in the lakes remain functionally adapted to metal contamination, potentially playing a crucial role in ecosystem recovery and bioremediation. These microorganisms could be leveraged to monitor and mitigate the effects of heavy metal contamination. Despite years having passed since the disaster, the microbiota of the lakes remains significantly impacted, reinforcing the need for continued research to fully understand and mitigate long‐term ecological consequences.

## Graphical abstract

The collapse of the Fundão dam caused long‐term alterations in the microbiome of lakes in the Doce River basin, with microbial communities closely associated with heavy metals originating from the mining tailings. The findings also highlight potential microbial groups for biomonitoring and bioremediation efforts.

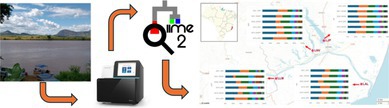

## 1 Introduction

On 5 November 2015, Brazil witnessed the largest environmental disaster in Latin America and the global mining industry, when the rupture of the Fundão and Santarém Dams in Mariana (MG) released about 55 million cubic metres of iron ore tailings into the Doce River (de Freitas et al. 2019). The tailings contained iron (Fe), silicon dioxide (SiO_2_), aluminium (Al), and traces of chromium (Cr), cadmium (Cd), and lead (Pb) (Escobar 2015), which are potentially toxic to human health, even at low doses (Balali‐Mood et al. 2021). The Doce River is one of the major watercourses in southeastern Brazil, providing water supply for more than 3 million people in several cities, as well as food since fishing is a common activity (SAMARCO 2016). Besides the river, the tailings reached the Atlantic Ocean, affecting important areas for biodiversity conservation, including the Abrolhos Marine National Park, which contains the largest and richest coral reefs of the Southern Atlantic (Fernandes et al. 2022).

Previous studies have demonstrated that the rupture of the Fundão dam affected the microbial communities of rivers within the Doce River basin and nearby coastal areas (de Almeida et al. 2023; Fernandes et al. 2022). These microbial alterations reflect profound ecological impacts, such as reduced diversity, collapse of ecological interactions, and loss of critical functions in biogeochemical cycles. However, it remains poorly understood whether such alterations persist in lentic environments, such as the basin's lakes, even nearly a decade after the disaster (Garris et al. 2018).

Lake recovery may follow trajectories distinct from those observed in rivers or marine areas, as these environments have unique ecological and hydrological characteristics, such as lower water renewal, greater accumulation of contaminated sediments, and high physicochemical stability. These factors can prolong pollutant impacts and hinder the recovery of microbial communities (Oliveira et al. 2018).

The primary route through which mining tailings impact microorganisms is via the toxicity of heavy metals. These elements directly interfere with microbial cell integrity, enzymatic metabolism, and community structure, favouring resistant species while eliminating sensitive ones. This process can reduce microbial diversity, simplify ecological networks, and compromise essential ecosystem functions, such as organic matter degradation and biogeochemical cycles—especially nitrogen cycling (Guo et al. 2019; de Almeida et al. 2023). Recent studies show that metals such as zinc, copper, cadmium, and lead can inhibit key enzymatic activity, alter microbial gene expression, and damage DNA, directly affecting the stability of microbial communities (Guo et al. 2019). Furthermore, the continued presence of these elements in environments impacted by mining tailings poses an on‐going threat to microbial ecological functionality, prolonging toxic effects over time (Ahemad 2019). Despite their ecological importance, microbial recovery in lakes affected by mining disasters remains understudied. This gap prevents a comprehensive understanding of long‐term impacts and the effectiveness of remediation efforts.

Considering these persistent and complex impacts on microbial communities, the objective of this study was to assess whether microbial communities in the lakes of the Rio Doce Basin remain altered in the long term, even several years after the Fundão dam collapse, the largest environmental disaster in Latin America.

To address this, we evaluated the composition, structure, and function of bacterial communities in four lakes of the basin between 2018 and 2021 using 16S rRNA sequencing and co‐occurrence network analysis.

## 2 Materials and Methods

### 2.1 Study Area, Samples Collection and Water Parameters Measurements

**FIGURE 1:**
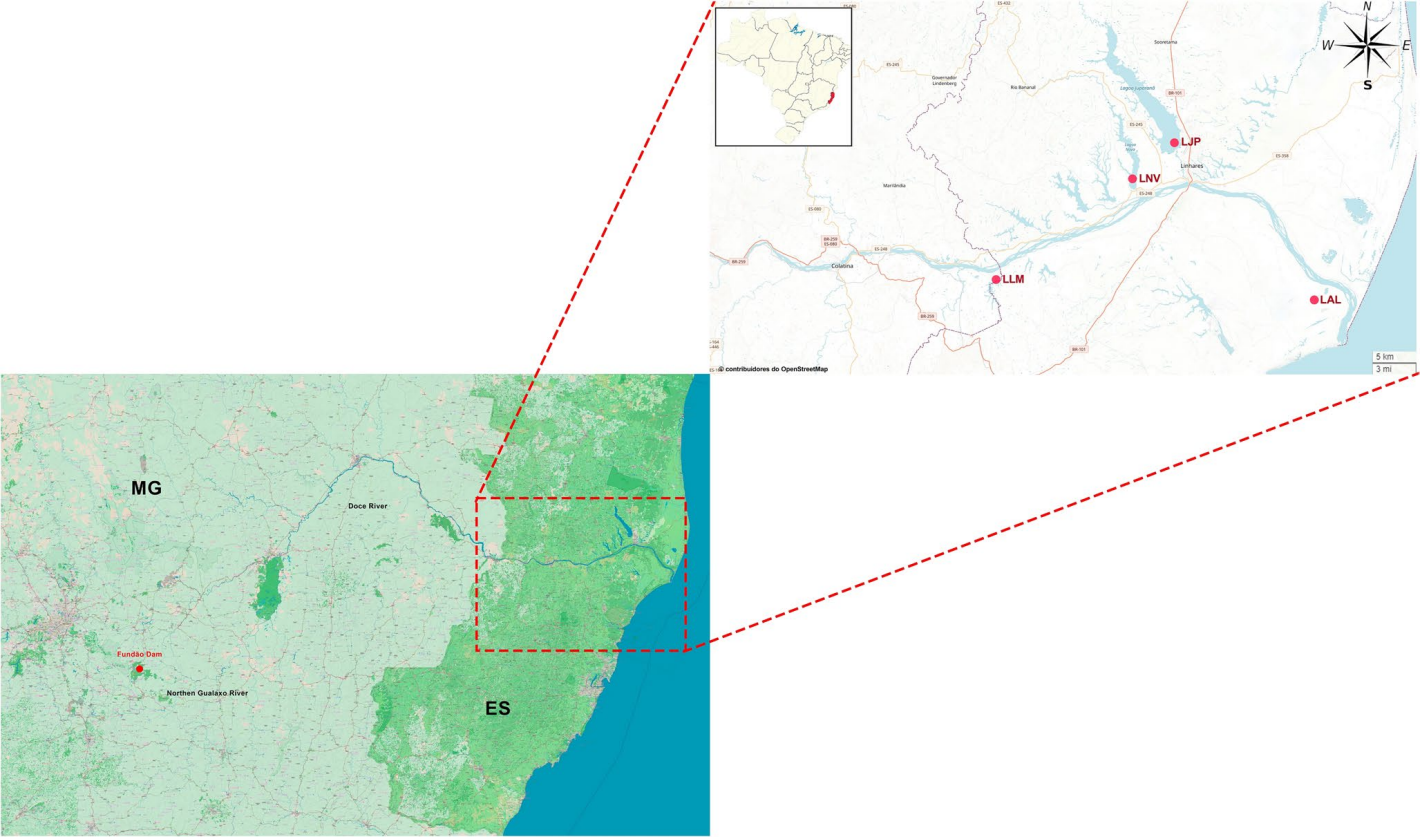
Coastal portion of the Doce River basin (Espírito Santo, Brazil). Red dots represent the sampling stations in Limão Lake (LLM), Lagoa Nova Lake (LNV), Juparanã Lake (LJP), and Areal Lake (LAL). The study area is delineated on the Brazil map in the insets on the top left.

Water and sediment samples were collected in triplicate from four lakes affected by mine sediment following the Fundão dam failure in Espírito Santo, Brazil. Sampling was conducted twice annually, during both the dry and rainy seasons, over a period of 4 years starting 3 years after the disaster. The collection period spanned from 2018 to 2021. Unfortunately, no samples were obtained during the 2020 rainy season due to the Covid‐19 pandemic quarantine. The lakes evaluated in this study were Limão Lake (LLM), Lagoa Nova Lake (LNV), Juparanã Lake (LJP), and Areal Lake (LAL) (Figure 1; Table S1). These lakes were selected because they are amongst the main water bodies impacted by the Fundão dam collapse. Located along the path of the tailings, they were directly affected, with contaminants accumulating in their waters and sediments. At Juparanã Lake, a barrier was constructed to prevent the entry of contaminated water from the Doce River. However, despite this containment measure, the lake was still affected, representing a secondary impact of the disaster.

Surface water samples (500 mL) were collected using a horizontal Niskin bottle and immediately filtered through 0.22 μm pore‐size nitrocellulose membranes. The filters were subsequently stored at −20°C. These procedures followed well‐established methodologies widely used in microbial ecology (Kaboosi et al. 2010; Odubanjo et al. 2021; De Oliveira et al. 2024). Sediment samples (10 g) were collected with a shovel or a Van Veen dredger, as appropriate, and also stored at −20°C. All samples were collected in triplicate, resulting in a total of 72 samples.

For each sample of water, we measured the physicochemical parameters (temperature, conductivity, salinity, pH, dissolved oxygen, dissolved organic carbon) using a multiparameter probe (YSI Professional Plus—Yellow Springs, Ohio, United States) submerged in 50 mL of water (Table S2). The data of these parameters in the samples obtained in 2018 and 2019 were retrieved from Almeida and colleagues (2023). Physicochemical variables for sediment were not measured.

### 2.2 DNA Extraction and Sequencing

DNA extraction was performed using the PowerSoil DNA isolation kit (Qiagen, USA) with 10 mL of each water sample and 0.5 g of sediment sample, following the manufacturer's protocol. Subsequently, DNA purity and concentration were assessed using the Qubit fluorometer (ThermoFisher—USA). The samples were then sent to the StarSEQ company (Mainz, Germany) for amplification and sequencing of the V4/V5 region of 16S ribosomal RNA (rRNA). PCR reactions utilised the 515F (GTGYCAGCMGCCGCGGTAA) and 909R (CCCCGYCAATTCMTTTRAGT) primers, employing the same temperature protocols previously described by Apprill et al. (2015) and Parada et al. (2016). Amplicon sequencing was carried out on an Illumina Miseq V3 platform (Illumina, USA) using a single‐end library.

### 2.3 Sequence Analysis

The raw data were demultiplexed and imported into the QIIME2 pipeline (Kuczynski et al. 2012). For time series comparison, we also imported the 16S reads from each lake previously described in (de Almeida et al. 2023) during the 2018 and 2019 rainy and dry seasons. The samples from 2020 and 2021 were collected as described in the previous chapter and were used exclusively in this study. The DADA2 plugin (Callahan et al. 2016) was utilised to assess read quality and cluster them into Amplicon Sequence Variants (ASVs). Taxonomic classification of each ASV was accomplished using the feature‐classifier plugin with a Bayesian classifier trained with the SILVA 138 database (Quast et al. 2012). Singletons, ASVs classified as Eukaryota and Archaea, off‐targets, and organellar DNA (chloroplast and mitochondria) were discarded. To assess sequencing depth adequacy, rarefaction curves were calculated.

### 2.4 Statistical Analysis

Alpha diversity analysis, including observed ASVs and Shannon index, (Ward et al. 1999; Shade 2017), was calculated using the R programming language (version 3.6.3) (R Core Team 2015).

For the beta diversity analysis, where we analysed the variation in the composition of the samples between locations of collections, we used the multidimensional scaling (MDS) (Wickelmaier 2003) obtained using the Phyloseq packages (McMurdie and Holmes 2013). The R software generated an ordering amongst all communities through the Bray‐Curtis distance. The differences between the bacterial profiles identified in each sample were analysed using the Kruskal‐Wallis (McKight and Najab 2010) and PERMANOVA tests, assuming *p* < 0.05.

Canonical Correspondence Analysis (CCA) (ter Braak 1986) was performed to establish the correlation between heavy metals, physicochemical parameters, and the abundance values of ASV identified as hubs. For this, we used the genus‐level ASV table with relative abundances as the first variable and the heavy metals table, based on data from Costa et al. (2022), as the second variable, using R software with the Vegan library (Oksanen et al. 2013) and ggplot2 packages (Wickham 2011). All data were normalised before statistical analysis and all statistical tests were performed considering significant values with a *p* value < 0.05.

### 2.5 Construction of the Co‐Occurrence Network

The network construction employed a set of nodes represented by the genera. We decided to use this level to avoid adding noise to the networks using the ASVs. Taxonomic levels higher than the genus could result in the loss of resolution. The edges connecting the nodes (Hu et al. 2017) denoted significantly positive or negative correlations between them. Co‐occurrence patterns in bacterial communities were assessed using the R program (version 3.6.3) (R Core Team 2015), utilising the relative abundance of the genera matrices as input data. The SparCC algorithm, capable of estimating Pearson's correlation values from compositional data (Friedman and Alm 2012), was utilised for co‐occurrence analysis. SparCC correlations with magnitudes greater than 0.4 or less than −0.4 were considered for the network analyses. The SpiecEasi library (Kurtz et al. 2015) in R was employed for generating the networks.

### 2.6 Hub Identification (Keystone Species)

Networks were analysed using the PageRank algorithm (Xing and Ghorbani 2004) to identify the key members of each microbiome. PageRank assesses the relevance of nodes based on link quantity and quality, assigning numerical weights that reflect their importance within the network. The nodes with the highest ranks, representing an average of the top 15 values for each microbiome, were selected as hubs in their respective networks.

### 2.7 Functional Prediction Using PICRUSt2

To predict the functional potential of microbial communities in the water and sediment samples, we employed the Phylogenetic Investigation of Communities by Reconstruction of Unobserved States version 2 (PICRUSt2) (Caicedo et al. 2020). PICRUSt2 was used to infer the abundance of gene families from 16S rRNA gene sequencing data.

## 3 Results and Discussion

In this study, we evaluated the long‐term impacts of the Fundão dam failure on the bacterial community dynamics and structure in the lakes of the Doce River basin, identifying keystone bacteria crucial for bioindication and bioremediation.

The sequencing of the V4/V5 region of the 16S rRNA yielded about 41 million prokaryotic reads, an average of 70,581 reads per sample. After quality control, 10 million reads were removed, resulting in an average of 53,000 reads per sample (Table S3). The rarefaction analysis indicated that all rarefaction curves reached their asymptotes (Figure S1).

Alpha diversity indices in our study were consistently higher in sediment samples than in water samples, as expected for benthic environments. Sediment richness ranged from 665 to 1682, while water samples ranged from 231 to 635 observed taxa (Figure S2). Although methodological differences between studies should be considered, Garris et al. (2018), who assessed microbial communities in lakes affected by the Mount Polley mine tailings spill in Canada, reported sediment richness values ranging from approximately 500 in highly impacted sites (Hazeltine Creek) to about 1500 in less impacted or unimpacted lakes (Quesnel, Polley, and Bootjack Lakes). This comparison suggests that the effects of mining tailings may persist for years after the initial disturbance. It is also important to note that the functional implications of changes in alpha diversity indices remain poorly understood (van Elsas et al. 2012).

**FIGURE 2:**
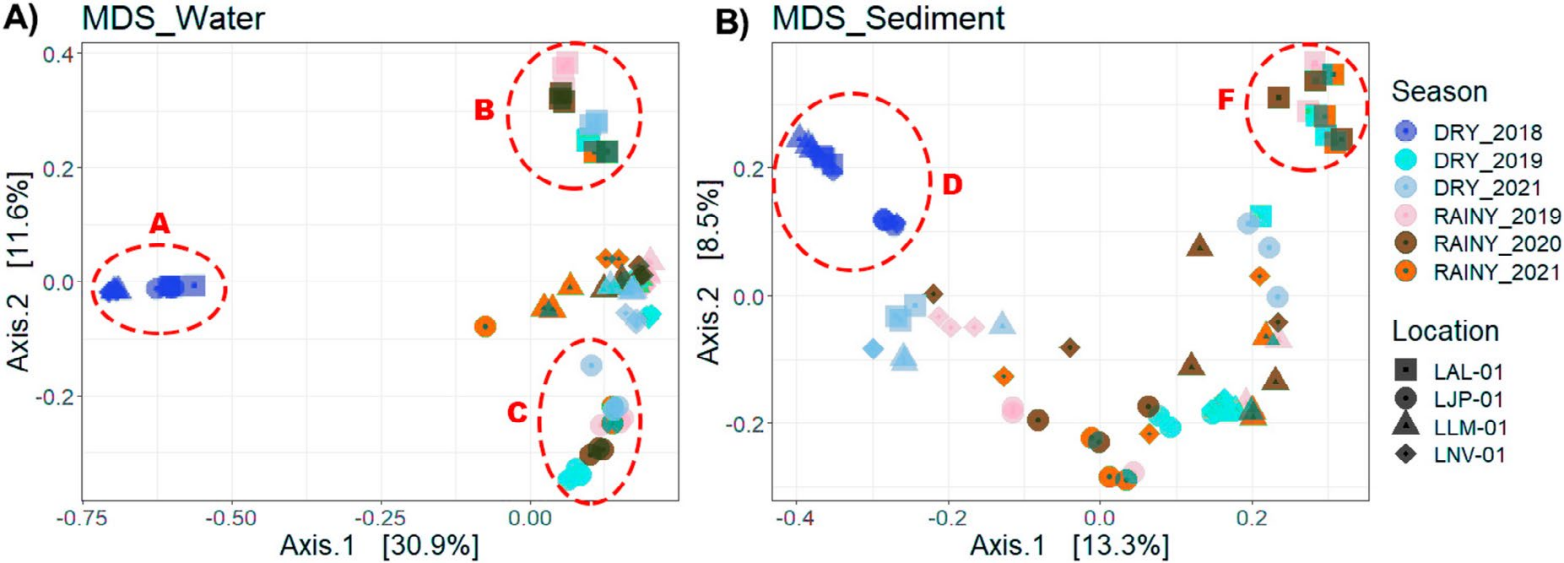
Multidimensional Scaling (MDS) ordination of bacterial ASVs based on Bray‐Curtis distances: (A) Water Samples, shows that the rainy 2019 and rainy 2020 samples cluster closer to each other along axis 1, explaining 30.9% of the variance. The 2018 drought and 2021 drought samples appear more centrally clustered. (B) Sediment Samples, shows that the rainy seasons of each year tend to cluster closer together along axis 1, explaining 13.3% of the variance. Samples campaigns are coloured according to the year and season: 2018 dry (yellow), 2019 dry (light brown), 2019 rainy (pink), 2020 rainy (green), 2021 dry (blue), 2021 rainy (dark brown). The square represents LAL, circle—LJP, LLM—triangle, LNV—diamond‐shape.

The MDS analysis of the bacterial community in the water and sediments of the lakes within the Doce River basin revealed a similar distribution in some aspects, influenced by seasonal variations and the isolation of certain lakes (Figure 2). The observed seasonal variability of the microbiome aligns with findings from previous studies (Bradshaw et al. 2020; Kjerfve and Magill 1989). Additionally, the differences in the microbiome amongst the lakes are expected, given the significant disparities between these distinct types of environments, which are typically unconnected (Kaevska et al. 2016).

**FIGURE 3:**
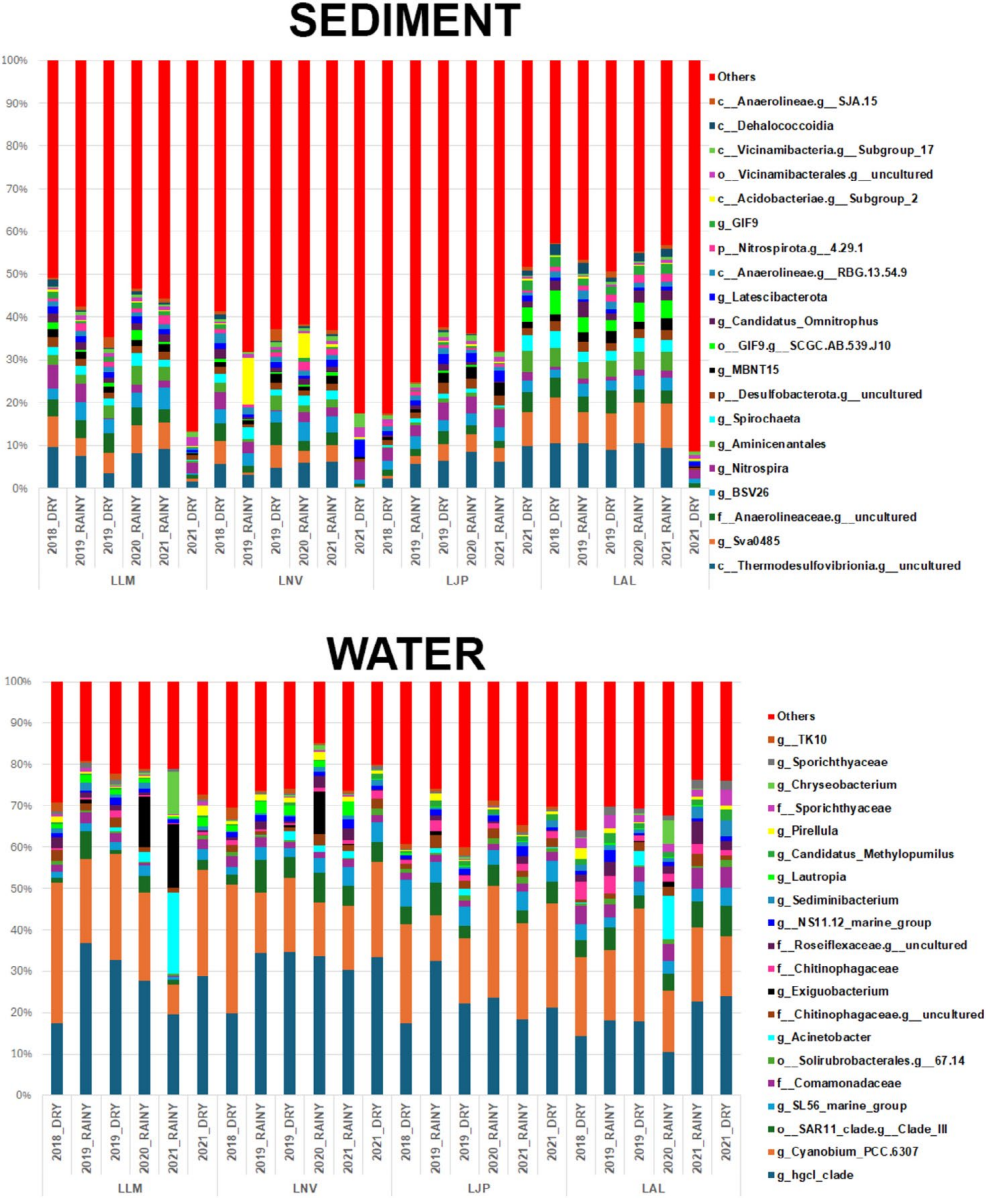
Relative abundance of microorganism taxa in water and sediment samples collected in different seasonal periods and years. (A) Relative abundance of microbial taxa in water samples collected in different seasonal periods and years. The main taxa are identified by name and colour. (B) Relative abundance of microbial taxa in sediment samples collected in different seasonal periods and years. The main functional categories are identified by name and colour. X: Time (years). Y: Relative abundance (%).

The profile of the bacterial community at the phyla level exhibited a predominance of Actinobacteriota, Cyanobacteria, Proteobacteria, Bacteroidota, and Chloroflexi as the most abundant phyla (Figure S3). Overall, the relative abundance of phyla remained consistent across all seasons, including both dry and rainy periods, although some temporal and regional variations were observed in response to different environmental conditions. Cyanobacteria showed low abundance in the Rio Doce basin immediately after the Fundão dam collapse (Cordeiro et al. 2019; Reis et al. 2020). This scarcity can be attributed to their photosynthetic characteristics, which depend directly on light penetration in the water—a factor severely compromised by the intense turbidity recorded in the days following the disaster (Reis et al. 2020). In particular, *Cyanobium_PCC.6307*, a unicellular cyanobacterium of great ecological importance (Coutinho et al. 2016; Sánchez‐Baracaldo et al. 2014), with global distribution and a key role in primary productivity (Callieri 2017; Fujimoto et al. 2015) was not detected in the impacted rivers in the days following the disaster (Reis et al. 2020), likely due to the high turbidity. At the genus level in the water (Figure 3A), a consistent pattern was observed, with *hgcI_clade* and *Cyanobium_PCC.6307* being the most abundant genera, with small changes at specific sites such as LLM Rainy 2020 and 2021, LNV Rainy 2020 and LAL Rainy 2021. At these points, an increase in the genera *Acinetobacter* (in LLM Rainy 2020 and 2021, and LAL Rainy 2021) and *Exiguobacterium* (in LLM Rainy 2020 and 2021 and LNV Rainy 2020).

The *hgcI clade* was predominantly identified in rivers impacted just a few days after the Fundão dam collapse (Reis et al. 2020) and had previously been reported as abundant in water bodies affected by acid mine drainage (Ettamimi et al. 2019). Unlike *Cyanobium_PCC.6307*, whose presence depends on favourable photosynthetic conditions, the high abundance of the *hgcI clade* reinforces the hypothesis that the disaster's effects continue to significantly influence the microbiome of these environments.

Other highly abundant bacterial taxa in water samples, such as *Sediminibacterium* and *Sporichthyaceae*, were also detected in the rivers shortly after the collapse (Reis et al. 2020). The persistence and high abundance of these groups 3 years after the disaster further support the evidence that the aquatic microbial community is still experiencing the prolonged effects of contamination.

In the sediment samples (Figure 3B), a recurring pattern was also observed, with the classes Thermodesulfovibrionia and *Sva0485*, as well as the family Anaerolineaceae, standing out as the most abundant in almost all sampling sites, except for LLM Dry 2021 and LAL Dry 2021 (Figure 3B). The Anaerolineaceae family has been reported as a core member of microbial communities in sediments contaminated by mining tailings (Chung et al. 2019), and has also been identified as the most abundant taxon in several environments severely impacted by heavy metals (Meng et al. 2019). According to Fernandes et al. (2022), this group was also predominant in marine areas affected by the Fundão dam tailings, particularly at the Doce River estuary. Its high abundance may be related to its ability to reduce heavy metals in syntrophy with archaea, possibly acting as a key microorganism for the bioremediation of these contaminated environments. *Nitrospira* was identified as the most abundant genus in sediments from tropical streams historically contaminated by metals, in a study conducted in the Iron Quadrangle region of Minas Gerais, the same area where the Fundão dam is located (Costa et al. 2022), recognised as one of the largest mining regions in the world (SAMARCO 2016). This finding suggests that *Nitrospira* may be part of the microbiota associated with the dam tailings, remaining in high abundance in the Doce River years after the disaster. Finally, the bacterial distribution patterns observed in the sediment samples were consistent with previous findings in freshwater ecosystems, including rivers belonging to the Doce River basin (Reis et al. 2020; Zhang et al. 2015).

**FIGURE 4:**
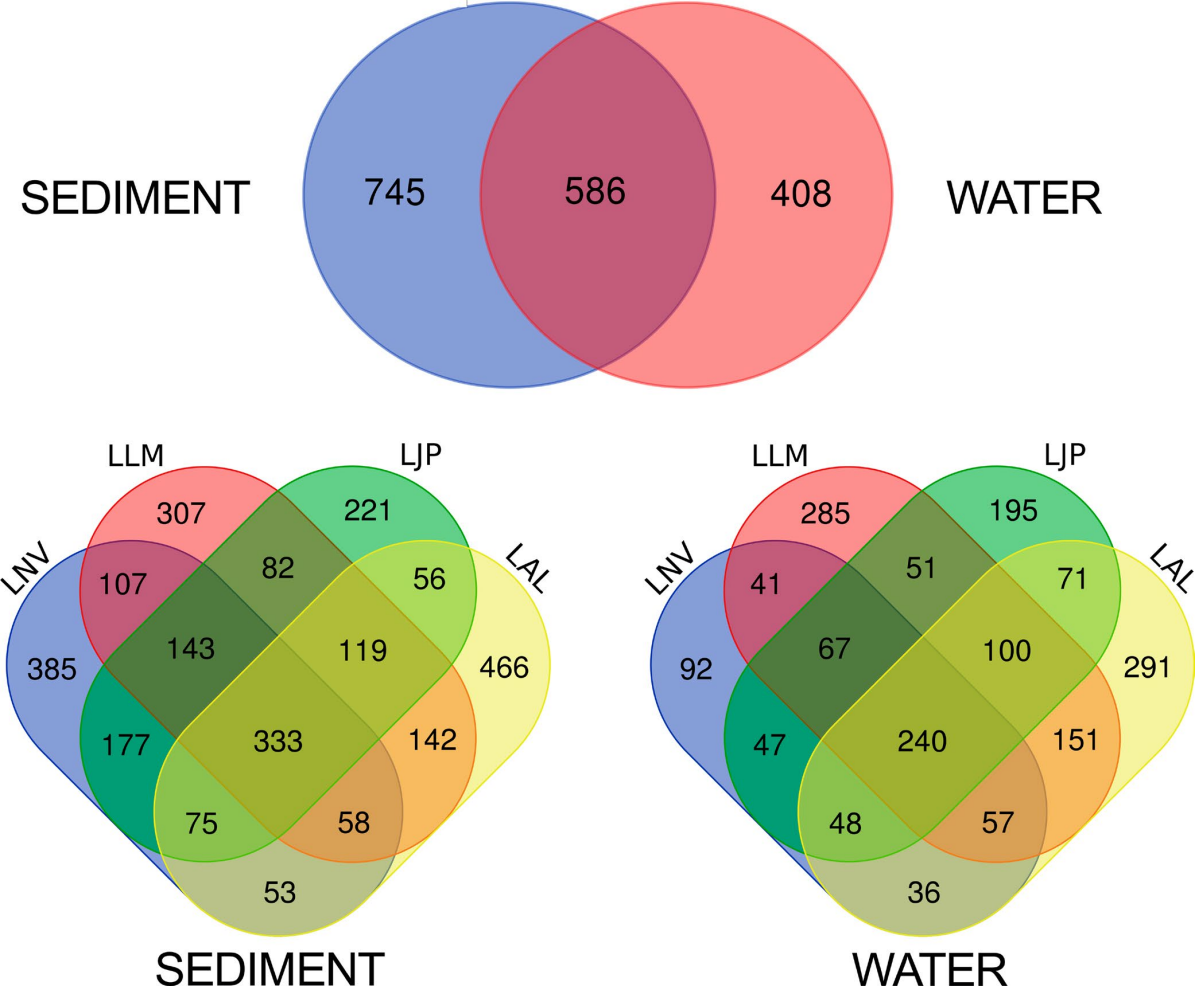
Venn Diagram: Where we have the areas of sharing and specificity of ASVs for each environment. (A) We have ASV's by type of environment; (B) The ASV's for each lagoon, for the sediment samples; (C) The ASV's for each lagoon, for the water samples.

The Venn Diagram analysis reveals interesting patterns in the distribution of ASVs across both water and lake sediment samples (Figure 4). In the water samples, a high number of ASVs is shared amongst all lakes, suggesting a degree of similarity in their aquatic microbiota. However, there is a high proportion of ASVs exclusive to specific sampling stations, as observed at the “LAL” station. This suggests that local factors or unique characteristics of this lake may influence the microbiota composition in a distinctive way (Bradshaw et al. 2020; Kjerfve and Magill 1989).

On the other hand, in the sediment samples, we found an even greater number of ASVs shared between the lakes, suggesting greater homogeneity in the sedimentary microbiota. However, unlike water samples, sediment samples show a more balanced distribution of lake‐specific ASVs between different water bodies. This may indicate that the composition of sediment microbiota can be influenced by local factors, but of a more uniform shape between the lakes (Bradshaw et al. 2020; Kjerfve and Magill 1989).

These results suggest that both the water and sediments of each lake have their own distinct microbiological characteristics, influenced by local factors and possibly by the interaction between different components of the aquatic ecosystem. This highlights the importance of considering not only water but also sediment when studying the microbial ecology of aquatic systems (Bradshaw et al. 2020; Kjerfve and Magill 1989).

**FIGURE 5:**
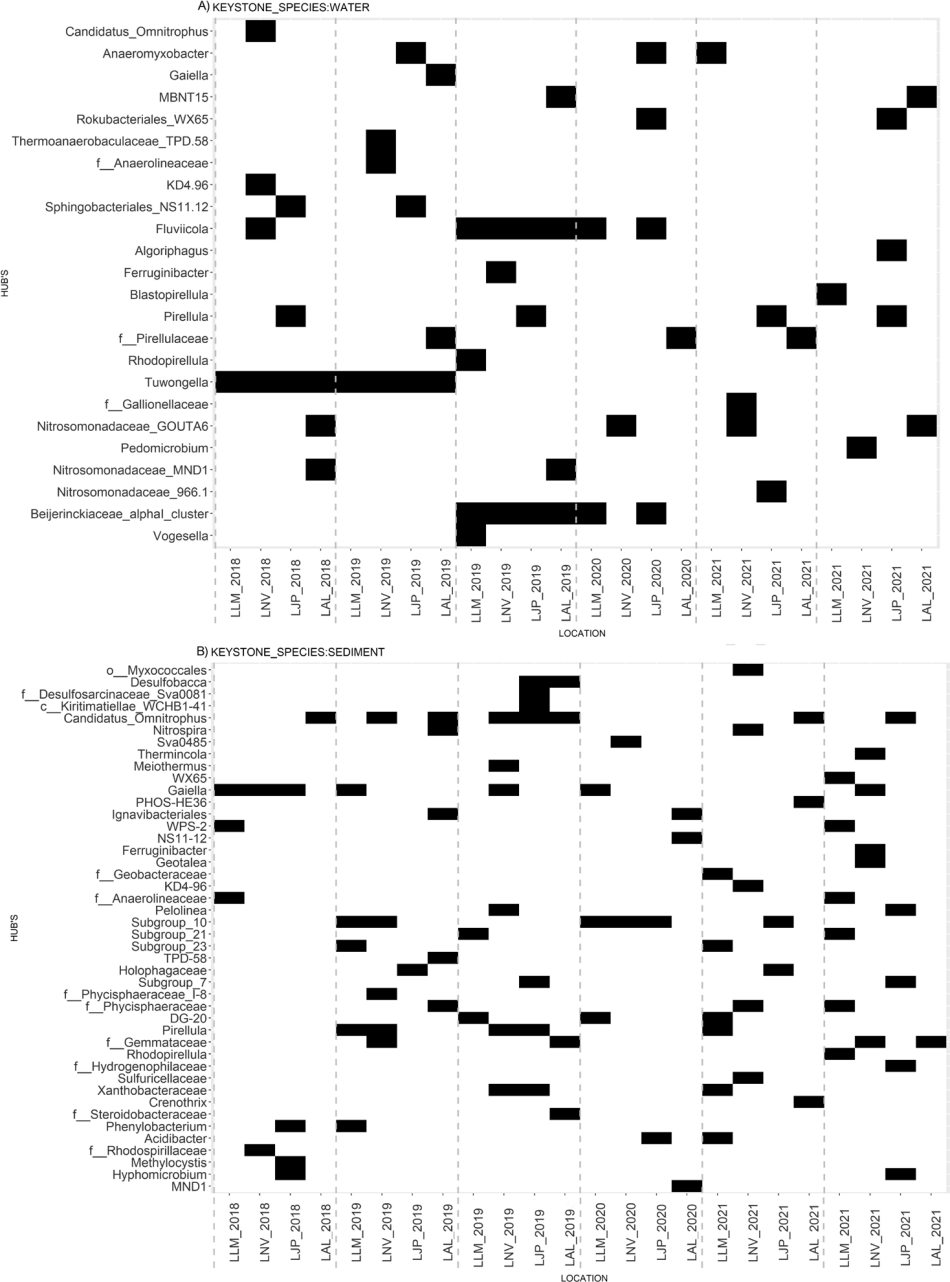
Hubs Identification: Each hub is represented by its location and season, with presence indicated by black colour and absence by white colour.

**FIGURE 6:**
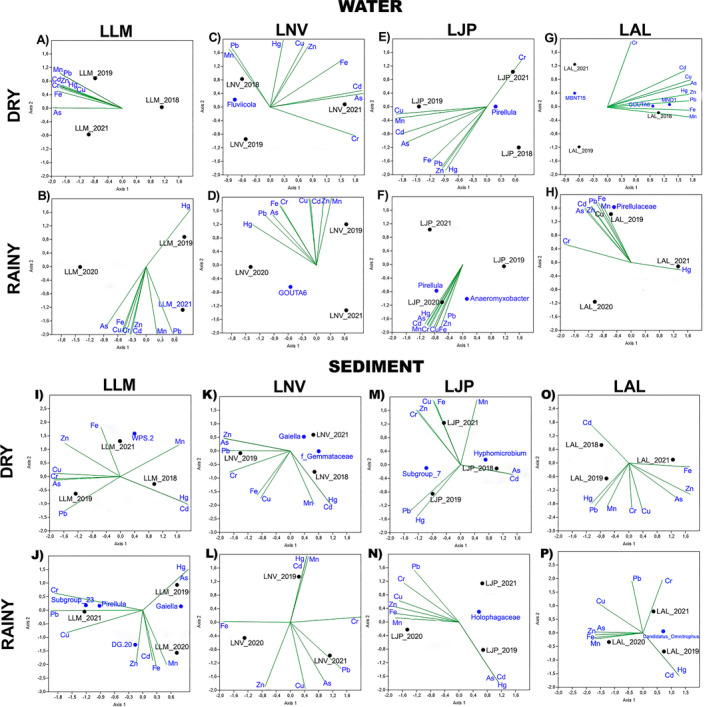
Canonical Correlation Analysis performed using the identified Hub ASVs, Water and Sediment, as the biotic variable and heavy metals, which include: Chromium (Cr), Lead (Pb), Iron (Fe), Manganese (Mn), Mercury (Hg), Zinc (Zn), Cadmium (Cd), Arsenic (As) and Copper (Cu).

To determine whether the keystone bacterial species in water and sediment are associated with metals and dam waste, co‐occurrence networks (Figures S4 and S5) were constructed (Table S4), and keystone species were identified (Figure 5). Moreover, Canonical Correspondence Analysis (CCA) was performed (Figure 6) to evaluate the effects of heavy metals and physicochemical properties on the identified key species. Based on these analyses, several keystone microorganisms associated with metals and dam waste were observed across all samples. Amongst these microorganisms, we highlight: *WPS‐2*, *Rokubacterales*, Thermoanaerobaculaceae *subgroup 2*3, genus *Pirellula*, *Acidibacter*, Geobacteraceae, *DG20*, Rhodospirillaceae, *Meiothermus*, family Anaerolineaceae, *SVA0485*, *KD4‐96*, *Nitrospira*, *Hyphomicrobium*, *Fervidicella*, *Subgroup 7 Holophagae*, Holophagaceae, *Sideroxydans*, and *MND1*. Many of these microorganisms were not identified as the most abundant in the samples analysed; only through network analysis and using the detection of hubs (keystone species) with the *pagerank* algorithm were we able to obtain these results.

The genera *WPS‐2* and *Rokubacterales* were detected in soils rich in iron and sulphur, and were positively correlated with iron, manganese, and zinc (Sheremet et al. 2020). The genus *Rhodopirellula*, present in both sediments and water, contains genes for resistance to heavy metals, aiding in the reduction of these metals in high concentrations (Chen et al. 2018; Lage et al. 2012; Øvreås et al. 2025). The Thermoanaerobaculaceae family, *subgroup 23*, demonstrated the ability to reduce iron and manganese, standing out as a potential bioremediator for mining waste (Dedysh et al. 2020; Kristensen et al. 2021).

The genus *Pirellula*, found in several lakes and periods, showed resistance to copper, suggesting its potential as a resilient species in contaminated environments (Lage et al. 2012; RRDM 2019). The genus *Gaiella* has been identified with high prevalence and considered a bioindicator of heavy metals (Yang et al. 2022). *Acidibacter* and the Geobacteraceae family are microorganisms capable of reducing iron minerals and act as bioindicators of heavy metal pollution. *DG20* is correlated with iron, selenium, and caesium, reinforcing its potential as an environmental indicator (Falagán and Johnson 2014; Röling 2014; Lusa and Bomberg 2021).

The Rhodospirillaceae family includes members resistant to metals such as iron and cobalt, in addition to degrading lead and cadmium, making it useful for bioremediation of contaminated areas (Röling 2014; Lusa and Bomberg 2021). The genus *Meiothermus* has shown the ability to reduce chromium, a highly toxic metal (Lv et al. 2018). The Anaerolineaceae family has been identified in mining tailings and contaminated environments and is known to degrade petroleum hydrocarbons and heavy metals (Chung et al. 2019; Liang et al. 2015).

The genus *SVA0485* was identified in sites with acid mine drainage, performing sulfate reduction, iron respiration, and fermentation under anaerobic conditions (Tan et al. 2019). The genus *KD4‐96*, belonging to the phylum Chloroflexi, was found in soils contaminated with iron, aluminium, and cadmium (Kujala et al. 2018; Liu et al. 2020). *Nitrospira*, an abundant genus in sediments, is essential in the nitrogen cycle and was identified as one of the most prevalent in environments contaminated by the Fundão dam disaster (de Almeida et al. 2023).

The genus *Hyphomicrobium* is capable of oxidising iron and manganese, while *Fervidicella* can reduce iron, manganese, and cobalt, playing important roles in environmental bioremediation (Vayenas 2011; Ogg and Patel 2010; Manav Demir 2016; Park et al. 2018). The *subgroup 7 Holophagae* and the family Holophagaceae are correlated with iron mining tailings and soil pH variations (Kielak et al. 2016; da Silva et al. 2022). The genus *Sideroxydans* acts in the formation of bioavailable iron oxides and in the biogeochemical cycle of iron, being essential in contaminated environments (Hädrich et al. 2019). The genus *MND1*, found in soils rich in iron and manganese, can be used as a bioindicator of contamination (Albuquerque et al. 2011; Yang et al. 2022).

**FIGURE 7:**
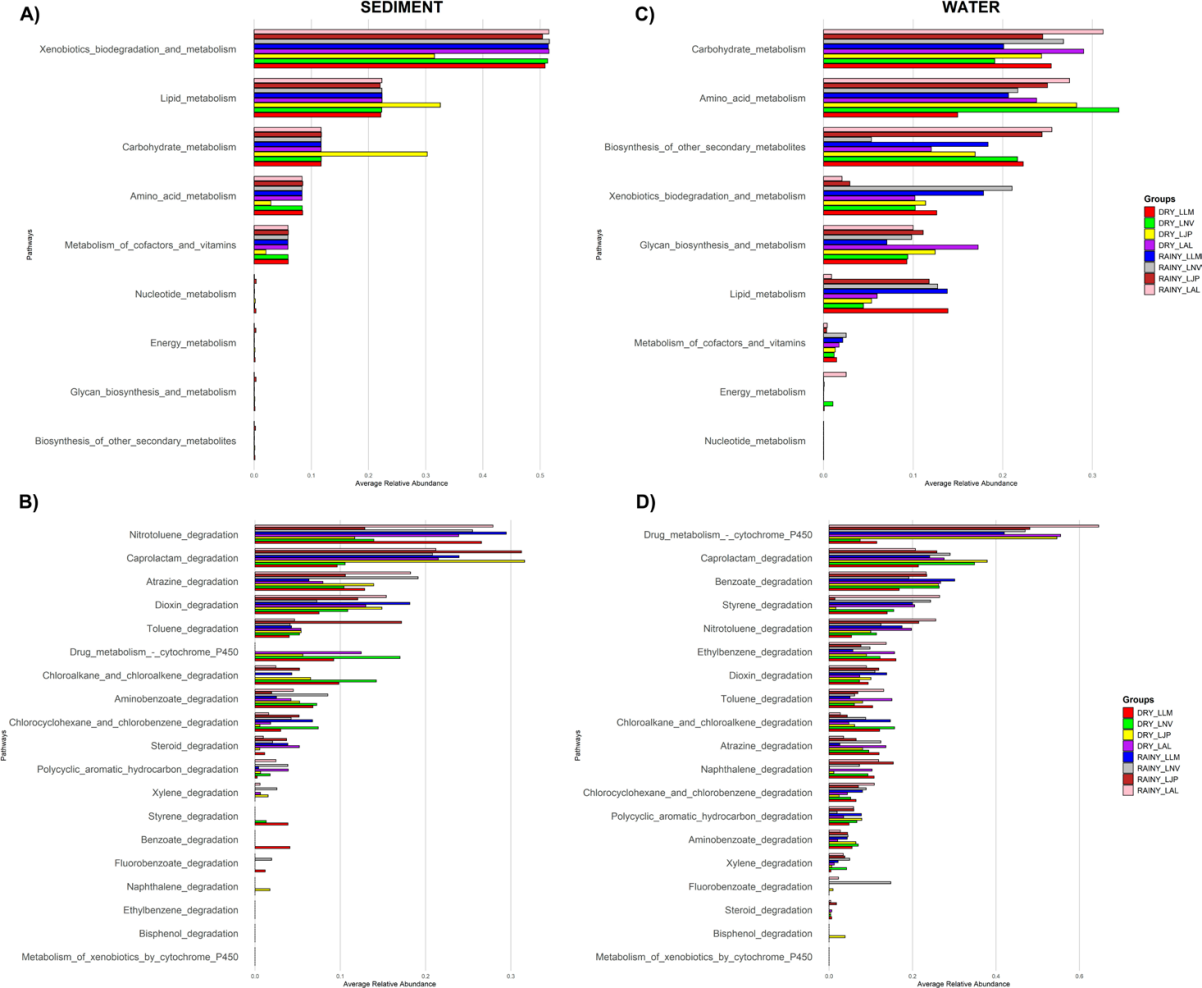
Relative abundance of metabolic pathways in water and sediment samples. Bar graphs represent the mean relative abundance of metabolic pathways in microbial communities from water (right panel) and sediment (left panel) samples collected at four sites (LAL, LJP, LNV, and LLM) during the dry (DRY) and rainy (RAINY) seasons over multiple years. The x‐axis shows the mean relative abundance, and the y‐axis lists the identified metabolic pathways. Colour‐coded bars indicate sampling groups: DRY_LLM (green), DRY_LNV (yellow), DRY_LJP (light green), DRY_LAL (red), RAINY_LLM (blue), RAINY_LNV (purple), RAINY_LJP (brown), RAINY_LAL (pink). (A) Level 2 metabolic pathways related to general metabolism in sediment. (B) Level 3 metabolic pathways related to biodegradation of xenobiotics in sediment. (C) Level 2 metabolic pathways related to general metabolism in water. (D) Level 3 metabolic pathways related to biodegradation of xenobiotics in water.

The analysis based on PICRUSt2.0 results shows a relevant relationship between microbial species and their predominant metabolic pathways under various environmental conditions (Figure 7). In the sediment samples, the metabolic pathway related to xenobiotics biodegradation and metabolism was extremely elevated, representing the most abundant pathway with an average relative abundance of 48.78%. In the water samples, xenobiotics biodegradation and metabolism were also highly abundant, although less so than in the sediment. It ranked as the fourth most abundant pathway, with an average relative abundance of 11.04% (Figure 7A,C). This abundance of xenobiotic degradation genes is commonly found in mining sediments (Chung et al. 2019). The Level 3 metabolic pathways related to xenobiotics biodegradation exhibited a high abundance of pathways associated with heavy metal metabolism or the metabolism of other compounds that co‐occur with heavy metals (Figure 7B,D).

In aquatic environments, the predominant pathways related to heavy metal detoxification include drug metabolism—cytochrome P450, mechanisms involving redox transformations, and metal chelation. These pathways reflect microbial adaptations to pollution stress and their role in the transformation and detoxification of xenobiotics and heavy metals (Copley 2009; Falkowski et al. 2008; Firincă et al. 2023).

In sediment data, pathways such as nitrotoluene degradation, chloroalkane and chloroalkene degradation, and polycyclic aromatic hydrocarbon (PAH) degradation were prominent. These pathways are frequently linked to heavy metal resistance due to their involvement in redox reactions and the metabolism of persistent pollutants often associated with metals (Kimes et al. 2014; McGenity et al. 2012; Ding et al. 2024).

Other notable pathways include caprolactam degradation, which reflects the ability to metabolise industrial compounds, and benzoate degradation, associated with the transformation of aromatic compounds often co‐occurring with heavy metals. Additionally, styrene degradation and atrazine degradation are relevant, as they involve mechanisms that can indirectly contribute to the detoxification of metal‐associated pollutants (Rojo 2009; Falkowski et al. 2008; Ding et al. 2024). These findings align with previous studies that demonstrated the enrichment of microorganisms capable of metabolising hydrocarbons and other complex organic compounds in environments impacted by mining activities (Fuchs et al. 2011; Kimes et al. 2014; Zhang et al. 2024; Zhu et al. 2025).

**FIGURE 8:**
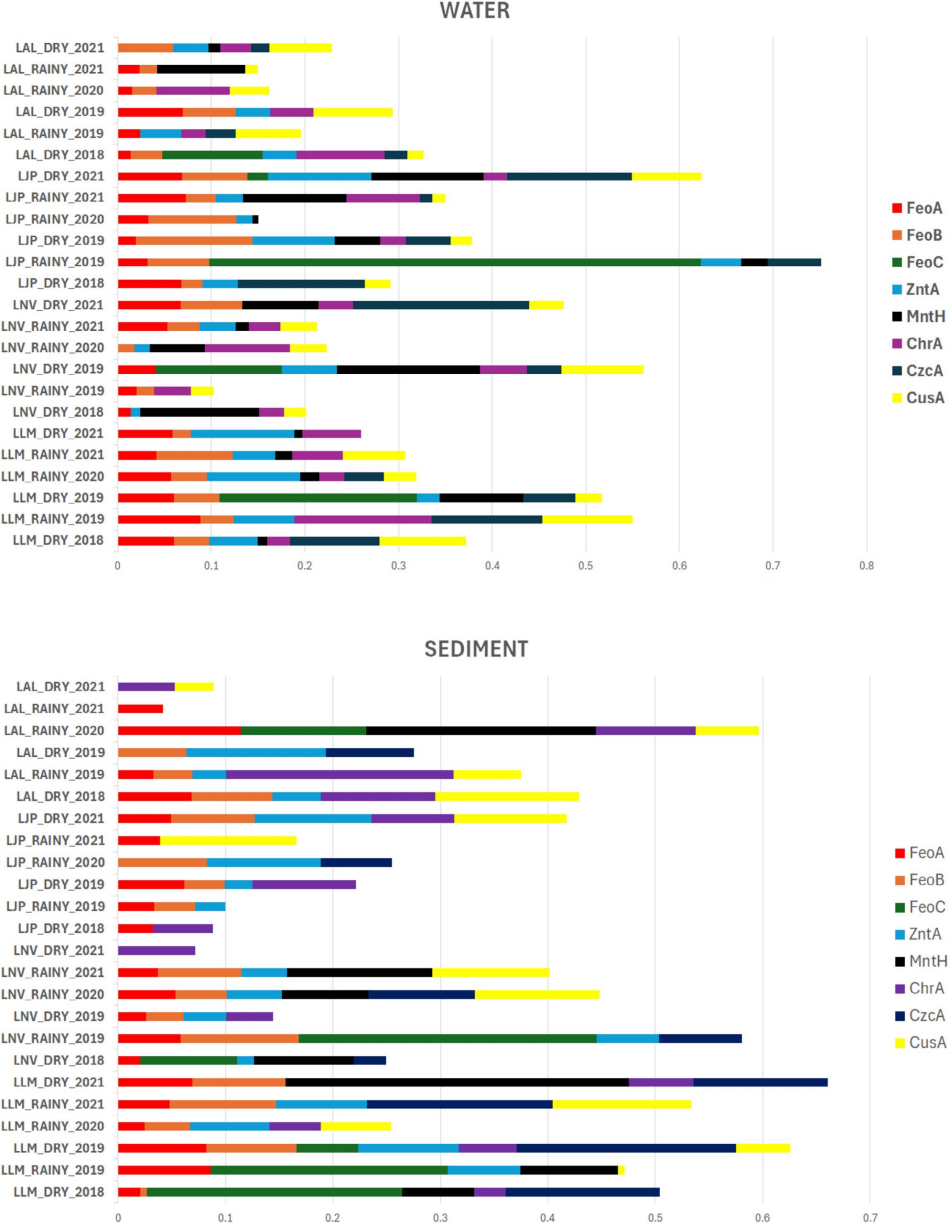
Relative abundance of associated genes in sediment and water samples from impacted environments. The figure highlights the prevalence of genes related to iron transport (FeoA, FeoB, and FeoC), manganese transport (MnTH), and heavy metal resistance (CusA, CzcA, and ZntA) across different sampling periods and locations. The bars indicate mean relative abundance (%) with error bars representing standard deviation. Sampling sites and conditions (e.g., dry or rainy seasons) are indicated for contextual analysis.

This pattern suggests that the microbial communities in both sediments and water are highly adapted to environments containing heavy metals and other associated contaminants, likely originating from the Fundão dam tailings. Moreover, in both sediment and water samples from these lakes, a high number of genes related to specific metals were also observed, including those associated with iron transport (FeoA, FeoB, and FeoC), manganese transport (MntH), and heavy metal resistance (CusA, CzcA, and ZntA) (Figure 8). Amongst the identified genes, those associated with iron, manganese, and zinc metabolism exhibited the highest abundance. The presence of these genes in microorganisms reveals a clear pattern of microbial adaptation to environments impacted by heavy metals (Desai et al. 2012; Gómez‐Garzón et al. 2022; Lau et al. 2013; Xiao et al. 2020; Kehres and Maguire 2003; Papp‐Wallace and Maguire 2006; Zheng et al. 2024; Cervantes et al. 2001; Andrei et al. 2020; Besaury et al. 2013; Dai et al. 2019).

Furthermore, several microbial hubs identified in this study in sediment and water may display these genes. In sediments, the FeoA and FeoB genes, associated with ferrous iron (Fe^2+^) transport, have been identified in several bacterial taxa such as *Rhodospirillaceae*, *Geobacteraceae*, *Ferruginibacter*, *Pirellula*, *Algoriphagus*, and *Desulfobacca* (Munk et al. 2011; Methé et al. 2003; Sun et al. 2025; Lim et al. 2009; Clum et al. 2009; Muraguchi et al. 2016; Göker et al. 2011). Recent studies reinforce the functional relevance of these genes across diverse bacteria. For instance, Gómez‐Garzón and Payne (2023) demonstrated the essential roles of *feoA* and *feoB* in *Helicobacter pylori* , highlighting their regulation in response to iron and nickel availability. Likewise, Sestok and colleagues (2022) characterised a *feoA–feoB* fusion protein in *Bacteroides fragilis* , revealing that FeoA is critical for stabilising the GTP‐bound form of FeoB, essential for iron transport under anaerobic conditions. These findings underscore the importance and evolutionary conservation of the Feo system in microbial iron acquisition.

The FeoC gene, involved in the regulation of iron uptake, was previously described in the Rhodospirillaceae family (Munk et al. 2011; Sestok et al. 2018; Hsueh et al. 1871) and the Geobacteraceae family (Methé et al. 2003; Sun et al. 2025). The MnTH gene, associated with manganese transport, was previously described in Hyphomicrobium (Brown et al. 2011), Methylocystis (Dam et al. 2012), Algoriphagus (Muraguchi et al. 2016), and Beijerinckiaceae (Tamas et al. 2010). The ZntA, CusA, and CzcA genes, related to zinc, copper, and cadmium resistance, were previously described as predominant in Algoriphagus (Han et al. 2017; Muraguchi et al. 2016; Nedashkovskaya et al. 2004), the Gallionellaceae family (Kojima et al. 2021), Geotalea (Prakash et al. 2010), and Vogesella (Gu and Cheung 2001; Sheu et al. 2016).

In water, similar patterns were observed; FeoA and FeoB genes were previously described in Fluviicola (Woyke et al. 2011), Pirellula (Clum et al. 2009), Blastopirellula (Storesund et al. 2018), and Anaeromyxobacter (Hwang et al. 2015). The FeoC gene was previously described in Algoriphagus (Muraguchi et al. 2016), emphasising its role in iron uptake under fluctuating environmental conditions. The MnTH gene, involved in manganese transport, was previously described in Methylocystis (Dam et al. 2012) and Hyphomicrobium (Brown et al. 2011). The CusA and CzcA genes, associated with heavy metal resistance, were previously described and detected in Rhodopirellula (Glöckner et al. 2003), Geotalea (Prakash et al. 2010), Algoriphagus (Han et al. 2017), and the Geobacteraceae family (Methé et al. 2003).

The enrichment of xenobiotic degradation pathways, together with evidence of metabolism associated with heavy metals such as iron, manganese, and zinc, along with the identification of several microbial hubs in this study capable of harbouring genes related to metal metabolism, highlights the continued adaptive responses of microbial communities in these lakes to heavy metal contamination, even years after the dam failure.

## 4 Conclusion

This study provides clear evidence that the microbial communities in lakes of the Doce River basin remain altered nearly a decade after the Fundão dam collapse. The persistent enrichment of microbial taxa such as *Deinococcus, Rhodospirillaceae, Pirellula, Ferruginibacter, Geobacteraceae, Algoriphagus, Desulfobacca, Hyphomicrobium, Methylocystis, Vogesella*, and *Anaerolineaceae*, many of which are functionally linked to metal metabolism and/or commonly found in environments severely impacted by heavy metals or mining tailings, indicate a long‐term shift likely driven by continued exposure to contaminants associated with mine tailings.

Additionally, the consistent detection of functional genes involved in metal transport and resistance, such as FeoA/FeoB (iron transport), FeoC (iron uptake regulation), MnTH (manganese transport), and ZntA, CusA, CzcA (zinc, copper, and cadmium resistance), highlights the metabolic adaptation of these microbial communities to contaminated environments.

These findings demonstrate not only the enduring ecological impact of the disaster but also underscore the potential of specific microbial taxa as bioindicators of contamination and as candidates for bioremediation. Continued monitoring of these freshwater systems is essential, as microbial indicators reveal that ecological recovery remains incomplete and environmental stress persists.

## Author Contributions

**Pedro Almeida:** data curation, formal analysis, visualization, writing – original draft, methodology, investigation, writing – review and editing, validation. **André Torres:** data curation, formal analysis, writing – review and editing, software, supervision. **Marcelos Gomes:** data curation, formal analysis, writing – review and editing, supervision, software. **Ernesto Caffarena:** methodology, formal analysis, writing – review and editing. **Hugo Jesus:** data curation, formal analysis, visualization, investigation, methodology, validation. **Pedro Pereira:** data curation, formal analysis, visualization, investigation, methodology, writing – review and editing. **Katariny Pereira Dos Santos:** methodology, data curation, validation. **Carlos Eduardo Delfino Vieira:** project administration, methodology. **Yuri Dornelles Zebral:** methodology, investigation. **Camila Martins:** methodology, supervision, project administration, conceptualization, investigation. **Adalto Bianchini:** conceptualization, methodology, investigation, supervision, project administration, writing – review and editing, funding acquisition, resources. **Henrique Santos:** conceptualization, data curation, formal analysis, validation, writing – review and editing, supervision, project administration.

## Conflicts of Interest

The authors declare no conflicts of interest.

## Supporting information


**FIGURE S1:** The rarefaction analysis performed on the microbial community data from sediment (left panel) and water (right panel) samples were collected from the Doce River basin. The x‐axis represents the sample size (number of sequences), and the y‐axis indicates the observed richness (number of distinct taxa). Each curve corresponds to a specific sample, showing the accumulation of taxa as sequencing depth increases. The asymptotic nature of the curves indicates that sequencing depth was sufficient to capture most of the microbial diversity within the samples, ensuring a comprehensive analysis.

**FIGURE S2:** This figure shows boxplots representing the observed richness (panels A and B) and Shannon diversity index (panels C and D) of microbial communities in water and sediment samples across different sampling stations (LAL, LJP, LLM, and LNV) and periods (dry and rainy seasons from 2018 to 2021). Panel A (Observed_Water). Panel B (Observed_Sediment). Panel C (Shannon_Water). Panel D (Shannon_Sediment). The colours represent different sampling stations:Red: LAL, Green: LJP, Blue: LLM, Purple: LNV.

**FIGURE S3:** Relative abundance of microorganism taxa in water and sediment samples collected in different seasonal periods and years. The microbial composition is presented at the phylum level, with the main taxa identified by name and corresponding colours. (A) Relative abundance of microbial taxa in water samples collected in different seasonal periods and years. The main taxa are identified by name and colour. (B) Relative abundance of microbial taxa in sediment samples collected in different seasonal periods and years. The main functional categories are identified by name and colour. X: Time (years). Y: Relative abundance (%).

**FIGURE S4:** Microbial co‐occurrence networks for water samples collected from four locations (LNV, LLM, LJP, LAL) across seasonal periods and years (2018–2021). Each network represents microbial associations at the ASV (Amplicon Sequence Variant) level. Nodes represent individual ASVs, colour‐coded by taxonomic classification. Edges indicate positive co‐occurrences (correlations) between ASVs. Columns: Sampling locations. Rows: Seasonal periods (Dry or Rainy) and corresponding years. The networks highlight spatial and temporal variations in microbial interactions within water ecosystems, illustrating the microbial community dynamics and connectivity influenced by environmental conditions and anthropogenic impacts.

**FIGURE S5:** Microbial co‐occurrence networks for sediment samples collected from four locations (LNV, LLM, LJP, LAL) across seasonal periods and years (2018–2021). Each network represents microbial associations at the ASV (Amplicon Sequence Variant) level. Nodes represent individual ASVs, colour‐coded by taxonomic classification. Edges indicate positive co‐occurrences (correlations) between ASVs. Columns: Sampling locations. Rows: Seasonal periods (Dry or Rainy) and corresponding years. The networks highlight spatial and temporal variations in microbial interactions within sediment ecosystems, illustrating the microbial community dynamics and connectivity influenced by environmental conditions and anthropogenic impacts.

**TABLE S1:** Sampling locations and their respective geographic coordinates. The table provides the identification code (ID), the name and location of each lake (Location), and the geographic coordinates (latitude and longitude) where the samples were collected. The sampling sites are located in the state of Espírito Santo, Brazil.

**TABLE S2:** Physicochemical properties of water samples collected from different lakes and sampling periods. The table includes data on sulphate (mg/L), chloride (mg/L), alkalinity (mg/L CaCO_3_), conductivity (μS/cm), dissolved oxygen (mg/L), pH, salinity (ppt), temperature (°C), and dissolved organic carbon (DOC, mg/L). The data were collected from four different lakes: Lagoa do Limão (LLM), Lagoa Nova (LNV), Lagoa Juparanã (LJP), and Lagoa do Areal (LAL) across multiple years (2018–2021) and seasons (dry and rainy).

**TABLE S3:** Sequence length statistics of the V4/V5 region of the 16S rRNA gene. The table summarises the sequence count, minimum length, maximum length, mean length, range, and standard deviation. Additionally, a seven‐number summary provides percentile values, indicating the distribution of sequence lengths across the data set. All values are rounded to the nearest whole number.

**TABLE S4:** Network analysis metrics of microbial communities in lake sediment and water samples under different seasonal and environmental conditions (2018–2021). The table summarises the number of positive edges, negative edges, total edges, and nodes in the microbial association networks for sediment and water samples. Positive and negative edges represent significant positive and negative correlations between microbial taxa, respectively, while total edges reflect the overall network connectivity. Nodes correspond to the number of microbial taxa present in the network. Results are presented for each condition, including different years, seasons (dry and wet), and collection sites (LNV, LLM, LJP, LAL). Sediment samples generally show higher connectivity (edges) and complexity (nodes) compared to water samples.

## Data Availability

The data that support the findings of this study are available on request from the corresponding author. The data are not publicly available due to privacy or ethical restrictions.
