# Supplementary figures and images for: Long‐Term Impact of the Largest Environmental Disaster in Latin America (Fundão Dam Failure) on Microbial Communities in Lakes of the Doce River Basin, Brazil
Source: Environ Microbiol. 2025 Sep 1;27(9):e70171. doi: 10.1111/1462-2920.70171 (PMC12400902; doi:10.1111/1462-2920.70171)

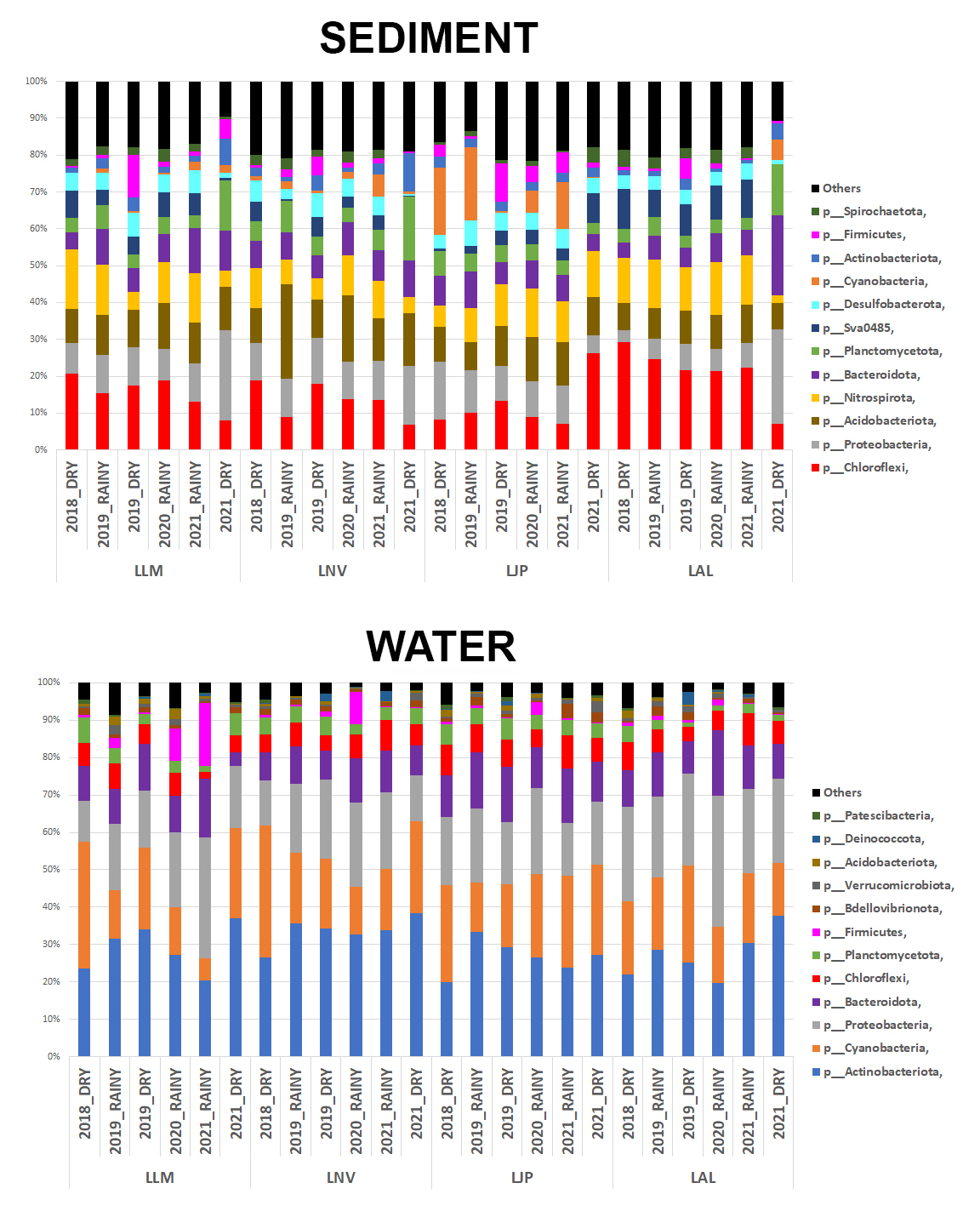

Supplement: Supplementary file 3 — FIGURE S3: Relative abundance of microorganism taxa in water and sediment samples collected in different seasonal periods and years. The microbial composition is presented at the phylum level, with the main taxa identified by name and corresponding colours. (A) Relative abundance of microbial taxa in water samples collected in different seasonal periods and years. The main taxa are identified by name and colour. (B) Relative abundance of microbial taxa in sediment samples collected in different seasonal periods and years. The main functional categories are identified by name and colour. X: Time (years). Y: Relative abundance (%). [file EMI-27-e70171-s003.jpg]

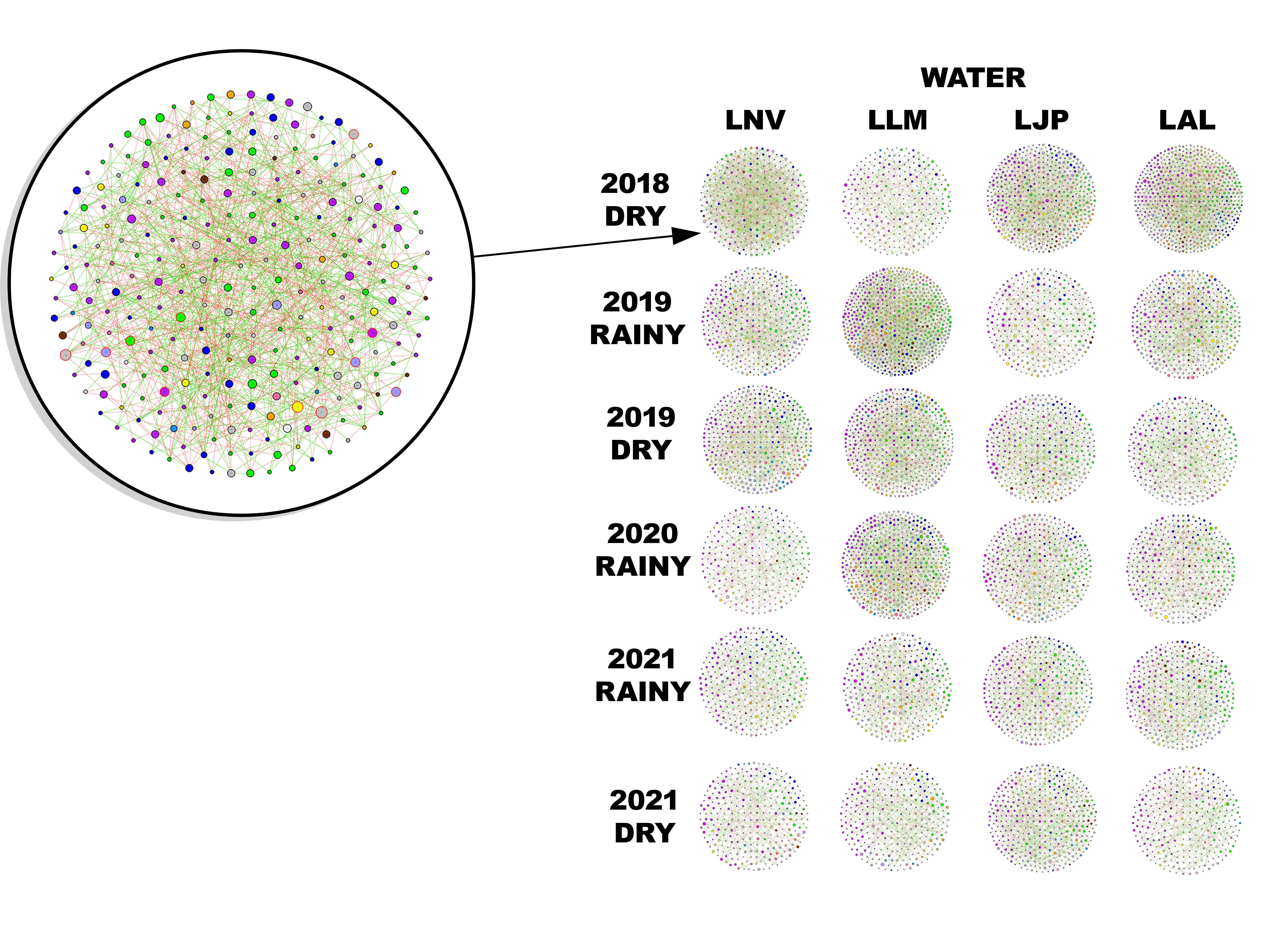

Supplement: Supplementary file 4 — FIGURE S4: Microbial co‐occurrence networks for water samples collected from four locations (LNV, LLM, LJP, LAL) across seasonal periods and years (2018–2021). Each network represents microbial associations at the ASV (Amplicon Sequence Variant) level. Nodes represent individual ASVs, colour‐coded by taxonomic classification. Edges indicate positive co‐occurrences (correlations) between ASVs. Columns: Sampling locations. Rows: Seasonal periods (Dry or Rainy) and corresponding years. The networks highlight spatial and temporal variations in microbial interactions within water ecosystems, illustrating the microbial community dynamics and connectivity influenced by environmental conditions and anthropogenic impacts. [file EMI-27-e70171-s004.jpg]

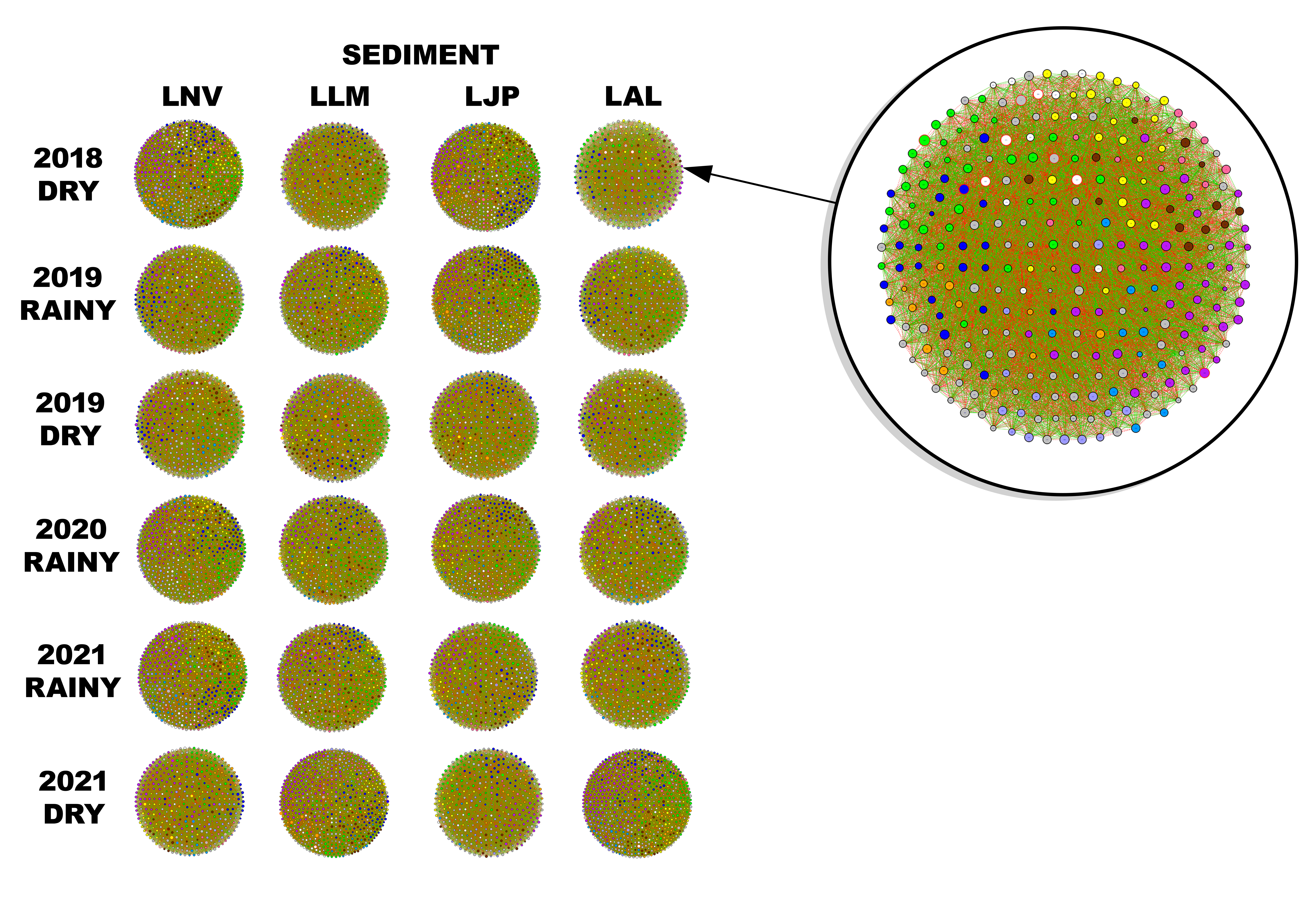

Supplement: Supplementary file 5 — FIGURE S5: Microbial co‐occurrence networks for sediment samples collected from four locations (LNV, LLM, LJP, LAL) across seasonal periods and years (2018–2021). Each network represents microbial associations at the ASV (Amplicon Sequence Variant) level. Nodes represent individual ASVs, colour‐coded by taxonomic classification. Edges indicate positive co‐occurrences (correlations) between ASVs. Columns: Sampling locations. Rows: Seasonal periods (Dry or Rainy) and corresponding years. The networks highlight spatial and temporal variations in microbial interactions within sediment ecosystems, illustrating the microbial community dynamics and connectivity influenced by environmental conditions and anthropogenic impacts. [file EMI-27-e70171-s002.jpg]
