# Supplementary material for: Long‐Term Impact of the Largest Environmental Disaster in Latin America (Fundão Dam Failure) on Microbial Communities in Lakes of the Doce River Basin, Brazil
Source: Environ Microbiol. 2025 Sep 1;27(9):e70171. doi: 10.1111/1462-2920.70171 (PMC12400902; doi:10.1111/1462-2920.70171)
Supplement: Supplementary file 6 — TABLE S1: Sampling locations and their respective geographic coordinates. The table provides the identification code (ID), the name and location of each lake (Location), and the geographic coordinates (latitude and longitude) where the samples were collected. The sampling sites are located in the state of Espírito Santo, Brazil. [file EMI-27-e70171-s008.docx]

#

# SUPPLEMENTARY TABLE 1:

| ***ID*** | ***Location*** | ***Geographic Coordinates*** | |
| --- | --- | --- | --- |
|  |  | **X** | **Y** |
| *LLM* | Lagoa do Limão - Colatina, ES | 19°39'01.7"S | 40°23'24.0"W |
| *LNV* | Lagoa Nova - Linhares, ES | 19°31'14.0"S | 39°47'08.8"W |
| *LJP* | Lagoa Juparanã - Linhares, ES | 19°16'58.9"S | 40°07'49.4"W |
| *LAL* | Lagoa do Areal - Linhares, ES | 19°35'07.7"S | 39°49'40.1"W |
