# Supplementary material for: Long‐Term Impact of the Largest Environmental Disaster in Latin America (Fundão Dam Failure) on Microbial Communities in Lakes of the Doce River Basin, Brazil
Source: Environ Microbiol. 2025 Sep 1;27(9):e70171. doi: 10.1111/1462-2920.70171 (PMC12400902; doi:10.1111/1462-2920.70171)
Supplement: Supplementary file 7 — TABLE S2: Physicochemical properties of water samples collected from different lakes and sampling periods. The table includes data on sulphate (mg/L), chloride (mg/L), alkalinity (mg/L CaCO3), conductivity (μS/cm), dissolved oxygen (mg/L), pH, salinity (ppt), temperature (°C), and dissolved organic carbon (DOC, mg/L). The data were collected from four different lakes: Lagoa do Limão (LLM), Lagoa Nova (LNV), Lagoa Juparanã (LJP), and Lagoa do Areal (LAL) across multiple years (2018–2021) and seasons (dry and rainy). [file EMI-27-e70171-s001.docx]

|  | *Sulfato* | *Chloride* | *Alkalinity* | *Conductivity* | *Dissolved O2* | *pH* | *Salinity* | *Temperature (°C)* | *Dissolved Organic Carbon(DOC)* |
| --- | --- | --- | --- | --- | --- | --- | --- | --- | --- |
| *LAL Dry 2018* | 9 | 6.79 | 26.44 | 136.3 | 6.7 | 6.64 | 0.07 | 28.7 | 12.31 |
| *LAL Rainy 2019* | 10.12 | 5.17 | 25.83 | 164.4 | 5.99 | 7.23 | 0 | 32.2 | 14.2 |
| *LAL Dry 2019* | 11.33 | 353.33 | 23.39 | 141.2 | 5.92 | 7.81 | 0.06 | 27 | 102.24 |
| *LAL Rainy 2020* | 8.09 | 141.33 | 14.04 | 0 | 0 | 0 | 0 | 0 | 31.45 |
| *LAL Rainy 2021* | 7.28 | 185.5 | 14.04 | 189 | 7.2 | 8.21 | 0.08 | 29.9 | 44.56 |
| *LAL Dry 2021* | 9.33 | 25.16 | 26.88 | 135.1 | 7.08 | 7.83 | 0.06 | 20.7 | 4301.33 |
| *LNV Dry 2018* | 6.5 | 7.4 | 36.61 | 88.5 | 7.9 | 7.02 | 0.04 | 26.9 | 2.23 |
| *LNV Rainy 2019* | 6.62 | 6.87 | 36.72 | 103.1 | 6.88 | 7.34 | 0 | 30.6 | 4.73 |
| *LNV Dry 2019* | 6.16 | 119.16 | 63.05 | 95 | 7.41 | 8.20 | 0.04 | 25.1 | 77.97 |
| *LNV Rainy 2020* | 4.40 | 47.66 | 37.83 | 85.2 | 6.4 | 6.79 | 0.04 | 29.9 | 23.98 |
| *LNV Rainy 2021* | 3.96 | 62.56 | 37.83 | 106 | 7.1 | 7.88 | 0.04 | 30.4 | 33.98 |
| *LNV Dry 2021* | 4.5 | 14.83 | 35.14 | 93.6 | 7.06 | 7.94 | 0.04 | 24.6 | 1429.16 |
| *LLM Dry 2018* | 6.33 | 9.70 | 32.54 | 110.3 | 7.4 | 7.03 | 0.06 | 25.7 | 3.90 |
| *LLM Rainy 2019* | 6.12 | 11.77 | 32.66 | 120.1 | 6.18 | 7.24 | 0 | 31.5 | 6.96 |
| *LLM Dry 2019* | 5.66 | 138.000 | 67.12 | 114.05 | 6.82 | 7.54 | 0.05 | 25.8 | 72.02 |
| *LLM Rainy 2020* | 4.05 | 55.2 | 40.27 | 96.3 | 6.9 | 7.3 | 0.05 | 31.7 | 22.15 |
| *LLM Rainy 2021* | 3.64 | 72.45 | 40.27 | 92.2 | 0 | 7.82 | 0.05 | 30.6 | 31.39 |
| *LLM Dry 2021* | 5.33 | 17.000 | 31.01 | 94.1 | 4.4 | 7.7 | 0.04 | 24.4 | 1491 |
| *LJP Dry 2018* | 6.66 | 7.4 | 32.54 | 85.3 | 7.9 | 7.15 | 0.04 | 25.3 | 4.26 |
| *LJP Rainy 2019* | 5.12 | 7.82 | 32.66 | 84.8 | 7.69 | 7.8 | 0 | 30.2 | 5.82 |
| *LJP Dry 2019* | 5.66 | 31.000 | 24.4 | 86.6 | 6.83 | 7.85 | 0.04 | 24.5 | 75.11 |
| *LJP Rainy 2020* | 4.04 | 12.4 | 14.65 | 93.9 | 7.34 | 8.36 | 0.04 | 29.3 | 23.11 |
| *LJP Rainy 2021* | 3.64 | 16.27 | 14.65 | 83 | 5.4 | 7.92 | 0 | 30.2 | 32.74 |
| *LJP Dry 2021* | 5.33 | 13.5 | 26.88 | 75.6 | 8.6 | 8.22 | 0.03 | 22.8 | 941.13 |

**Supplementary table 2:**

# 
