# Supplementary material for: Long‐Term Impact of the Largest Environmental Disaster in Latin America (Fundão Dam Failure) on Microbial Communities in Lakes of the Doce River Basin, Brazil
Source: Environ Microbiol. 2025 Sep 1;27(9):e70171. doi: 10.1111/1462-2920.70171 (PMC12400902; doi:10.1111/1462-2920.70171)
Supplement: Supplementary file 8 — TABLE S3: Sequence length statistics of the V4/V5 region of the 16S rRNA gene. The table summarises the sequence count, minimum length, maximum length, mean length, range, and standard deviation. Additionally, a seven‐number summary provides percentile values, indicating the distribution of sequence lengths across the data set. All values are rounded to the nearest whole number. [file EMI-27-e70171-s009.docx]

**Supplementary Table 3:**

| **Sequence Count** | **Min Length** | **Max Length** | **Mean Length** | **Range** | **Standard Deviation** |  |  |  |  |  |  |  |  |  |  |  |  |  |  |
| --- | --- | --- | --- | --- | --- | --- | --- | --- | --- | --- | --- | --- | --- | --- | --- | --- | --- | --- | --- |
| 114190 | 299 | 300 | 299.73 | 1 | 0.44 |  |  |  |  |  |  |  |  |  |  |  |  |  |  |
|  |  |  |  |  |  |  |  |  |  |  |  |  |  |  |  |  |  |  |  |
| **Seven-Number Summary of Sequence Lengths** | | | | | | | | | | | | | | | | | | | |
| **Percentile:** | **2%** | **9%** | **25%** | **50%** | **75%** | **91%** | **98%** |  |  |  |  |  |  |  |  |  |  |  |  |
| Length* (nts): | 299 | 299 | 299 | 300 | 300 | 300 | 300 |  |  |  |  |  |  |  |  |  |  |  |  |
| *Values rounded down to nearest whole number. | | | | | | | | | | | | | | | | | | | |
