# Supplementary material for: Long‐Term Impact of the Largest Environmental Disaster in Latin America (Fundão Dam Failure) on Microbial Communities in Lakes of the Doce River Basin, Brazil
Source: Environ Microbiol. 2025 Sep 1;27(9):e70171. doi: 10.1111/1462-2920.70171 (PMC12400902; doi:10.1111/1462-2920.70171)
Supplement: Supplementary file 9 — TABLE S4: Network analysis metrics of microbial communities in lake sediment and water samples under different seasonal and environmental conditions (2018–2021). The table summarises the number of positive edges, negative edges, total edges, and nodes in the microbial association networks for sediment and water samples. Positive and negative edges represent significant positive and negative correlations between microbial taxa, respectively, while total edges reflect the overall network connectivity. Nodes correspond to the number of microbial taxa present in the network. Results are presented for each condition, including different years, seasons (dry and wet), and collection sites (LNV, LLM, LJP, LAL). Sediment samples generally show higher connectivity (edges) and complexity (nodes) compared to water samples. [file EMI-27-e70171-s005.docx]

# Supplementary table 4:

| *SEDIMENT* | *positive edges* | *negative edges* | *edges* | *nodes* | *WATER* | *positive edges* | *negative edges* | *edges* | *nodes* |
| --- | --- | --- | --- | --- | --- | --- | --- | --- | --- |
| *DRY_2018_LNV* | 20550 | 21857 | 42407 | 624 |  | 299 | 282 | 581 | 269 |
| *DRY_2018_LLM* | 8253 | 8909 | 17162 | 392 |  | 67 | 73 | 140 | 276 |
| *DRY_2018_LJP* | 19807 | 21325 | 41132 | 615 |  | 237 | 239 | 476 | 478 |
| *DRY_2018_LAL* | 4128 | 4477 | 8605 | 278 |  | 283 | 281 | 564 | 558 |
| *RAINY_2019_LNV* | 10306 | 11105 | 21411 | 440 |  | 134 | 146 | 280 | 379 |
| *RAINY_2019_LLM* | 11790 | 12491 | 24281 | 468 |  | 352 | 305 | 657 | 595 |
| *RAINY_2019_LJP* | 17065 | 18375 | 35440 | 569 |  | 66 | 80 | 146 | 288 |
| *RAINY_2019_LAL* | 7868 | 8517 | 16385 | 384 |  | 156 | 149 | 305 | 396 |
| *DRY_2019_LNV* | 8263 | 8968 | 17231 | 397 |  | 166 | 161 | 327 | 418 |
| *DRY_2019_LLM* | 8251 | 9054 | 17305 | 394 |  | 211 | 196 | 407 | 460 |
| *DRY_2019_LJP* | 8650 | 9457 | 18107 | 406 |  | 144 | 119 | 263 | 346 |
| *DRY_2019_LAL* | 6606 | 7115 | 13721 | 353 |  | 115 | 98 | 213 | 319 |
| *RAINY_2020_LNV* | 16853 | 17917 | 34770 | 565 |  | 60 | 70 | 130 | 253 |
| *RAINY_2020_LLM* | 10745 | 11507 | 22252 | 448 |  | 236 | 221 | 457 | 492 |
| *RAINY_2020_LJP* | 13345 | 14273 | 27618 | 502 |  | 126 | 124 | 250 | 369 |
| *RAINY_2020_LAL* | 8877 | 9546 | 18423 | 406 |  | 105 | 116 | 221 | 338 |
| *RAINY_2021_LNV* | 18518 | 19808 | 38326 | 592 |  | 98 | 78 | 176 | 293 |
| *RAINY_2021_LLM* | 13405 | 14522 | 27927 | 506 |  | 98 | 94 | 192 | 321 |
| *RAINY_2021_LJP* | 9526 | 10228 | 19754 | 423 |  | 118 | 118 | 236 | 348 |
| *RAINY_2021_LAL* | 8949 | 9817 | 18766 | 411 |  | 122 | 113 | 235 | 351 |
| *DRY_2021_LNV* | 7910 | 8746 | 16656 | 388 |  | 79 | 77 | 156 | 282 |
| *DRY_2021_LLM* | 24179 | 26076 | 50255 | 678 |  | 91 | 84 | 175 | 296 |
| *DRY_2021_LJP* | 7419 | 8065 | 15484 | 373 |  | 132 | 121 | 253 | 374 |
| *DRY_2021_LAL* | 25811 | 27329 | 53140 | 700 |  | 68 | 65 | 133 | 279 |
